# Molecular docking study of nuciferine as a tyrosinase inhibitor and its therapeutic potential for hyperpigmentation

**DOI:** 10.5808/gi.23054

**Published:** 2023-09-27

**Authors:** Veerabhuvaneshwari Veerichetty, Iswaryalakshmi Saravanabavan

**Affiliations:** Department of Biotechnology, Kumaraguru College of Technology affiliated with Anna University, Coimbatore, Tamil Nadu 641006, India

**Keywords:** ascorbic acid, hyperpigmentation, kojic acid, melanin, nuciferine, resorcinol, tyrosinase inhibitors

## Abstract

Melanin is synthesized by tyrosinase to protect the skin from ultraviolet light. However, overproduction and accumulation of melanin can result in hyperpigmentation and skin melanoma. Tyrosinase inhibitors are commonly used in the treatment of hyperpigmentation. Natural tyrosinase inhibitors are often favoured over synthetic ones due to the potential side effects of the latter, which can include skin irritation, allergies, and other adverse reactions. Nuciferine, an alkaloid derived from *Nelumbo nucifera*, exhibits potent antioxidant and anti-proliferative properties. This study focused on the *in silico* screening of nuciferine for anti-tyrosinase activity, using kojic acid, ascorbic acid, and resorcinol as standards. The tyrosinase protein target was selected through homology modeling. The residues of the substrate binding pocket and active site pockets were identified for the purposes of grid box optimization and docking. Therefore, nuciferine is a potent natural tyrosinase inhibitor and shows promising potential for application in the treatment of hyperpigmentation and skin melanoma.

## Introduction

Nuciferine is a naturally occurring alkaloid found in the leaves and seeds of the sacred lotus (*Nelumbo nucifera*) plant. As an aporphine alkaloid, it shares structural similarities with other alkaloids present in plants. The sacred lotus has a rich history of use in Ayurvedic and Chinese medicine, with nuciferine recognized as one of its bioactive components possessing potential therapeutic properties. Numerous studies have explored the pharmacological activities of nuciferine, revealing several beneficial effects. One of its significant properties is its antioxidant activity, which aids in protecting cells from oxidative stress and damage inflicted by free radicals. Moreover, nuciferine has been examined for its potential role in managing obesity and associated metabolic disorders. It has shown the capacity to affect lipid metabolism, inhibit adipogenesis (the formation of fat cells), and decrease the accumulation of triglycerides [1]. These findings imply that nuciferine may hold promise as a natural agent for weight management. Additionally, nuciferine displays anti-inflammatory activity by suppressing pro-inflammatory mediators and enzymes. This trait suggests its potential in developing therapeutic interventions for inflammatory conditions [2,3]. Studies have also been conducted on nuciferine's anticancer properties. It has demonstrated the ability to induce apoptosis (programmed cell death) and inhibit the growth and proliferation of cancer cells [4,5]. This has sparked interest in investigating nuciferine as a potential anticancer agent. In summary, the diverse pharmacological activities of nuciferine make it a topic of considerable interest in various fields, including medicine, nutrition, and cosmetics. However, more research is required to fully comprehend its mechanisms of action and explore its potential applications.

Melanin, a pigment that determines the color of skin, hair, and eyes, plays a crucial role in shielding the skin from harmful ultraviolet (UV) radiation by absorbing and scattering UV photons. The enzyme tyrosinase is a central regulator of melanin production, participating in the melanin biosynthetic pathway [6]. Tyrosinase transforms the amino acid tyrosine into dopaquinone, which is subsequently processed to produce different types of melanin, including eumelanin (responsible for brown and black pigmentation) and pheomelanin (responsible for red and yellow pigmentation). Dysregulation or dysfunction of tyrosinase can result in various skin pigmentation disorders, such as hyperpigmentation (excess melanin) or hypopigmentation (insufficient melanin). The control of tyrosinase activity using tyrosinase inhibitors has attracted considerable interest in both scientific and commercial sectors. Tyrosinase inhibitors are substances that can disrupt the enzymatic activity of tyrosinase, thereby controlling melanin production. These inhibitors have potential uses in cosmetics, medicine, and agriculture. Cosmetic formulations frequently incorporate tyrosinase inhibitors to address hyperpigmentation issues, including melasma, age spots, and post-inflammatory hyperpigmentation. By diminishing the activity of tyrosinase, these inhibitors can reduce melanin production and foster a more uniform skin tone. Natural compounds such as arbutin, licorice extract, and kojic acid, along with synthetic agents like hydroquinone and its derivatives, are commonly employed as tyrosinase inhibitors in cosmetics [7]. The control of tyrosinase activity and melanin production also has implications in medicine. In the treatment of vitiligo, a condition marked by the loss of melanocytes and depigmented patches on the skin, tyrosinase inhibitors can stimulate re-pigmentation by encouraging melanin production. Moreover, inhibiting tyrosinase has demonstrated potential in certain cancer therapies, as melanin biosynthesis is vital for the growth and survival of melanoma cells. In agriculture, tyrosinase inhibitors are used to prevent enzymatic browning in fruits and vegetables. These inhibitors obstruct the activity of tyrosinase, preventing the oxidation of phenolic compounds and preserving the visual appeal and shelf life of perishable produce. This paper aims to provide a comprehensive examination of the interplay between tyrosinase inhibitors and melanin. It details the mechanisms of action of tyrosinase inhibitors, their impact on melanin production, and their applications in various fields [7].

The paper will also discuss the challenges and prospects associated with tyrosinase inhibitors, including potential side effects, regulatory aspects, and burgeoning research. By clarifying the connection between tyrosinase inhibitors and melanin, this paper enhances our comprehension of the fundamental mechanisms and potential uses of these inhibitors. It illuminates their multifaceted roles in skincare, medicine, and agriculture, thereby laying the groundwork for additional research and development in this field.

## Methods

### Exploring protein structure: secondary structure analysis and two-dimensional topology prediction

In this study, we retrieved the protein sequences of human tyrosinase from the NCBI database using the GenBank ID AAA61241.1 [8]. We conducted a BLASTp search to identify proteins with sequences similar to our target, using default parameters. This search was performed against the Protein Data Bank (PDB), and we did not exclude any organisms from the analysis. We selected the top hits from the BLAST results based on their significance, only considering sequences that shared a minimum identity of 44%. These selected human tyrosinase protein sequences serve as valuable references in our study, enabling comparisons and further analysis. By using the BLASTp algorithm, our search strategy ensured a comprehensive coverage of the protein database and considered a wide range of potential matches. This approach lays a solid foundation for subsequent investigations into the characteristics and functions of tyrosinase, and aids in enhancing our understanding of its role in various biological processes.

### Two-dimension topology prediction

The Protter tool (http://wlab.ethz.ch/protter) [9] was utilized to generate a two-dimensional topology representation of the protein. This tool enabled the visualization and mapping of the protein's structural elements onto a two-dimensional plane, thereby providing a clear illustration of its topological arrangement. This analysis yielded important insights into the connectivity and spatial organization of secondary structure elements, including alpha-helices and beta-sheets. These insights further enriched our comprehension of the protein's structural attributes and assisted in interpreting its functional implications.

### Comparative structure modeling of human tyrosinase and mushroom tyrosinase

The three-dimensional structure of hsTYR was determined using Modeller v9.25 software, which employs spatial restraints to guide the modeling process (https://salilab.org/modeller/) [10]. The amino acid sequence of the target hsTYR, EC 1.14.18.1 (GenBank ID: AAA61241.1), was converted into the ALI file format that Modeller can read. The modeling procedure consisted of four main steps. First, template recognition and selection were conducted by searching the PDB for proteins with known structures that are homologous to hsTYR. BLAST was used to compile a list of top hits, from which templates (PDB ID: 5M8Q, 5M8L) were selected. The template that had the highest identity to the target sequence was chosen, and its alignment with the target sequence was established. Finally, Modeller was used to generate five successful models of hsTYR based on the selected template structure. These models can be visualized using the SAVES Server. The three-dimensional structure of mushroom tyrosinase AbTYR (tyrosinase *Agaricus bisporus*, GenBank ID: ADE67053.1) was also determined using Modeller v9.25 software, using the same process.

### Protter: visualizing protein structures

Protter is a web-based tool designed to visualize protein sequences graphically. It generates annotations for protein features, including secondary structure elements, domains, and post-translational modifications, and presents these in an interactive and informative way. Users can upload a protein sequence or provide a UniProt identifier to receive a visual depiction of the protein's structure and functional elements. Protter offers a user-friendly method for exploring and analyzing protein sequences, assisting in the interpretation of their biological significance.

### Evaluation of the generated model:

The most robust theoretical structural models of hsTYR and AbTYR were validated through the examination of phi/psi torsion angle distributions in the protein structure, as analyzed by Ramachandran plot using the SAVES server. The stereochemical quality of the model was further scrutinized using the PROCHECK server, which computed several parameters. These included side chain conformations of protein structures as a function of atomic resolution, the geometry of hydrogen bonds, and the angles and planarity of peptide bonds.

### Selection of protein targets

The crystal structures of two proteins were retrieved from the PDB: the human tyrosinase-related protein 1 (TRP1) with the protein sequence PDB ID: 5M8L, and PPO3, a tyrosinase from *Agaricus bisporus*, with the inhibitor tropolone (AbTYR) and the protein sequence PDB ID: 2Y9W.

### Ligand preparation for melanin

The following ligands were sourced from PubChem: nuciferine (PubChem CID – 10146), kojic acid (PubChem CID – 3840), ascorbic acid (PubChem CID – 54670067), and isobutylamido thiazolyl resorcinol (PubChem CID – 71543007) [11-13]. The residues that form the binding pockets were identified through an extensive review of the literature.

### Molecular docking of nuciferine, thiazolyl resorcinol, kojic acid, and ascorbic acid in hyperpigmentation

Melanin plays a crucial role in skin protection. TYR and TYRP1 are significantly associated with the risk of malignant melanoma, which is responsible for most skin cancer-related deaths. The use of a tyrosine inhibitor has shown clinical benefits in the treatment of skin melanoma and associated local melanoma hyperpigmentation. In this study, we screened nuciferine for its potential to target the catalytic site of tyrosinase, thereby inhibiting abnormal melanogenesis. This could have therapeutic potential for skin melanoma [14,15]. We used Autodock Vina 4.2 for molecular docking [16,17], and BIOVIA Discovery Studio Visualizer for analyzing 2D and 3D interactions.

## Results

AutoDock Vina 4.2 was utilized for docking and the calculation of binding energies, as well as determining hydrogen bond interactions. Nuciferine was compared to vemurafenib, which served as the reference drug. Vemurafenib is commonly used as a standard drug for targeting the treatment of skin melanoma, particularly inhibiting BRAF proteins. Nuciferine demonstrates anticancer activity by inducing oxidative stress and halting cell progression, among other mechanisms. Unlike conventional antitumor medications, nuciferine provides a range of therapeutic benefits, including anti-inflammatory, anti-anxiety, and anti-cancer properties.

### Prediction of the 2-D structure of human TRP1

**Fig. 1.**
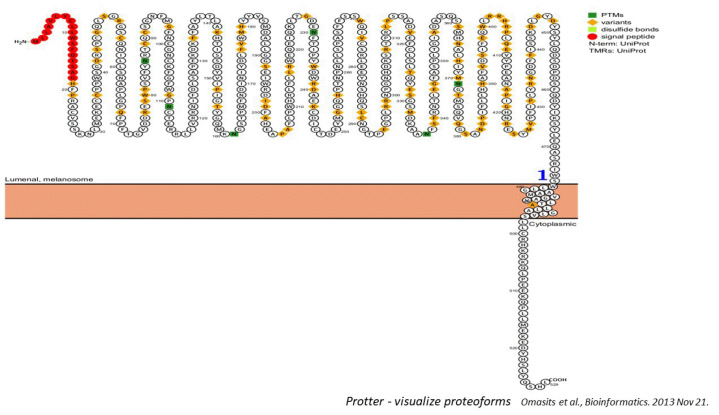
The two-dimensional topology and transmembrane prediction for TRP1 using PROTTER [1].

The first phase of our multi-stage strategy to model a 3D structure of human tyrosinase involved predicting its potential secondary structure and two-dimensional topology. We used the TRP1 protein sequence as the input for the Protter tool to accomplish this. As shown in Fig. 1, the Protter results predicted a graphical representation of a single transmembrane helix in TRP1, featuring a large extracellular and intracellular loop.

### 3D Homology modeling of AbTYR and TRP1 by Modeller

**Fig. 2.**
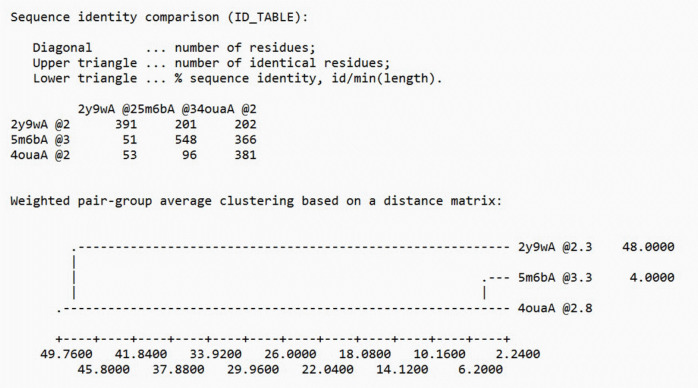
Template selection based on the sequence identity comparison table (AbTYR) by Modeller [10].

The specific mechanism of drug binding and transport by TRP1 remains largely unknown due to the absence of a solved crystal structure record [18,19]. We used an integrated approach to predict the 3D modeled structure of AbTYR, which included comparative modeling based on the satisfaction of spatial restraints using Modeller v.9.25 [20,21]. We selected the templates (PDB ID: 2y9w, 5m6b, 4oua) by conducting a BLAST search and scanning the PDB for proteins with determined structures that were homologous [22]. From these, we chose the best template (2y9wA), which had the highest identity with the target sequence. This selection was based on the sequence identity comparison table generated by Modeller, as depicted in Fig. 2.

**Fig. 3.**
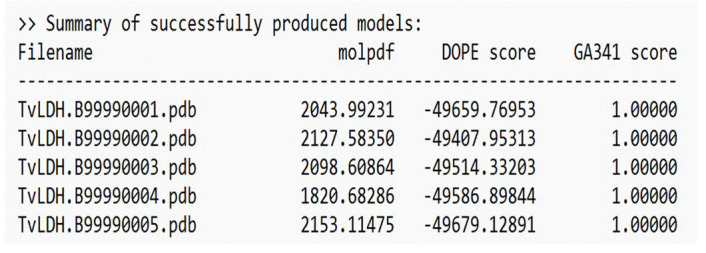
Models generated by Modeller (AbTYR) [10].

The selected template structure is that of the mushroom tyrosinase transporter (PDB ID: 2y9wA). After constructing a target-template alignment, Modeller automatically calculates a 3D model of the target using its auto model class. Five successful models of AbTYR were generated based on the 2y9wA template structure, as depicted in Fig. 3.

**Fig. 4.**
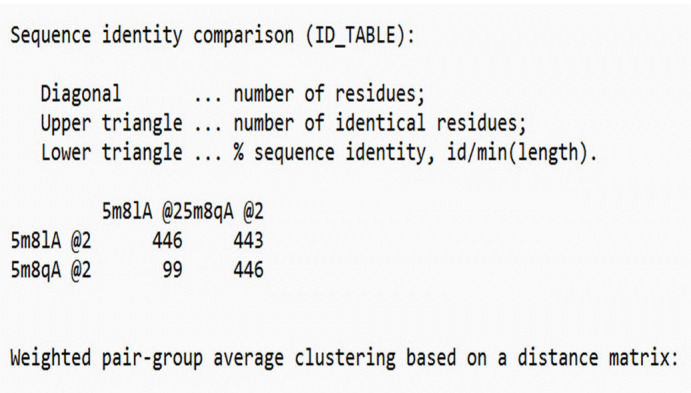
Template selection based on the sequence identity comparison table (TRP1) [10].

We used an integrated approach to predict the 3D modeled structure of TRP1, which included comparative modeling based on the satisfaction of spatial restraints using Modeller v.9.25. We selected the templates (PDB ID: 5M8L, 5M8Q) by conducting a BLAST search and scanning the PDB for proteins with determined structures that were homologous. We then chose the best template (5m8lA), which had the highest identity with the target sequence, based on the sequence identity comparison table generated by Modeller, as depicted in Fig. 4.

**Fig. 5.**
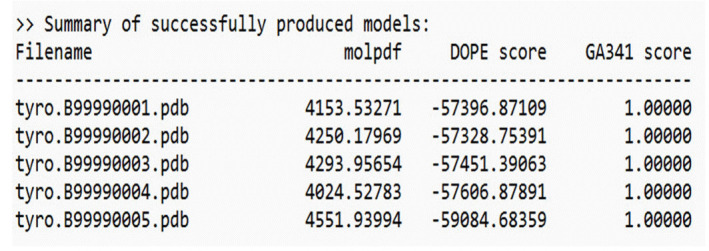
Models generated by Modeller (TRP1) [10].

The selected template structure was that of the mushroom tyrosinase transporter (PDB ID: 5m8lA). After a target-template alignment is established, Modeller automatically generates a 3D model of the target using its automodel class. Five successful models of TRP1 were created using the 5m8lA template structure, as shown in Fig. 5.

### Validation of the 3D-modeled structure of AbTYR and TRP1

The structural information of the mushroom tyrosinase protein plays an essential role in determining its biological function and transport mechanism. As such, validating the model of this protein has become an essential step in assessing its quality [23]. This quality assessment involves analyzing the Ramachandran plot via the SAVES server. A Ramachandran plot is a graphical representation that illustrates the conformational angles of amino acids within a protein structure, specifically showing the distribution of phi (ϕ) and psi (ψ) angles for each residue. This plot is instrumental in visualizing the permissible and impermissible regions of conformational space for the backbone torsion angles, thereby offering insights into the overall structural quality and stability of the protein. Through the analysis of a Ramachandran plot, it is possible to determine whether the modeled protein structure aligns with the anticipated protein backbone conformation.

**Fig. 6.**
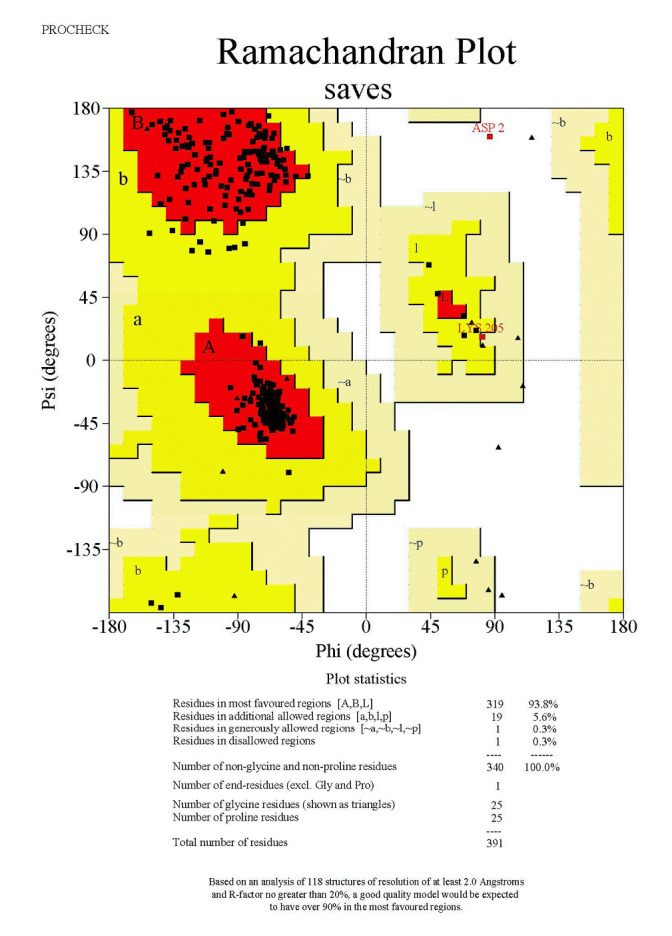
Ramachandran plot analysis of modelled structure of AbTYR.

Fig. 6 presents results from the SAVES server, indicating that the model contains 93.8% of residues in the most favoured regions, 5.6% of residues in additional allowed regions, and a mere 0.3% of residues in disallowed regions.

Due to the pivotal role of structural information in modeling the human tyrosinase protein, which is key to understanding its biological function and transport mechanism, it is critical to validate the model to assess its quality. This quality assessment involves analyzing the Ramachandran plot via the SAVES server.

**Fig. 7.**
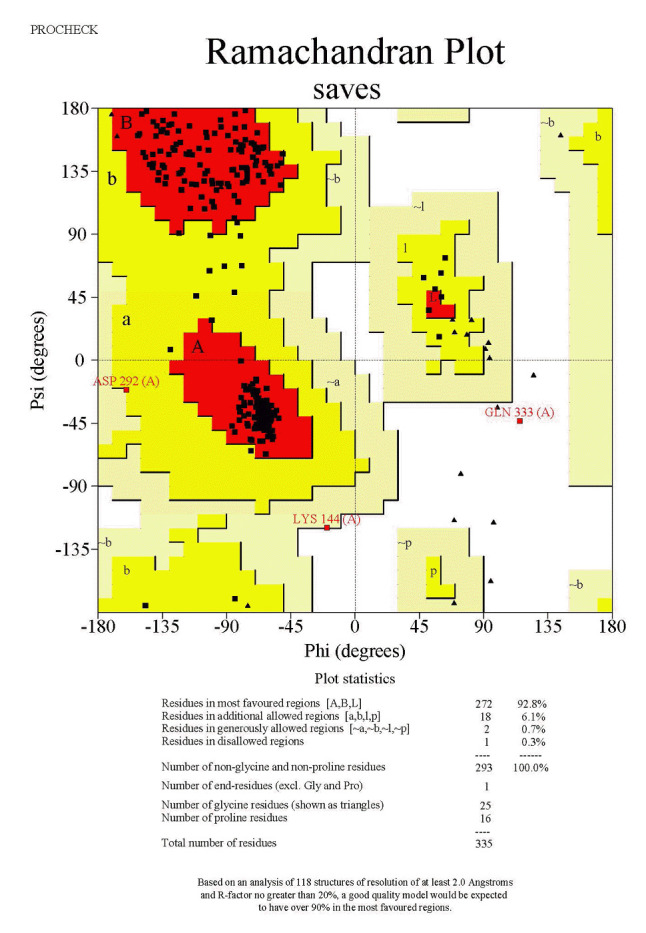
Ramachandran plot analysis of modelled structure of HsTYR.

As shown in Fig. 7, the results from the SAVES server indicate that the model contains 93.8% of residues in the most favoured regions, 6.1% of residues in additional allowed regions, and 0.3% of residues in disallowed regions.

### Ligand and protein retrieval and preparation for melanin

The proteins retrieved from the PDB included the crystal structure of human TRP1 with PDB ID (5M8L), and the crystal structure of PPO3, a tyrosinase from *Agaricus bisporus*, with inhibitor tropolone (AbTYR) and PDB ID (2Y9X). The ligands retrieved from PubChem included nuciferine with PubChem CID (10146), kojic acid with PubChem CID (3840), ascorbic acid with PubChem CID (54670067), and isobutylamido thiazolyl resorcinol with PubChem CID (71543007). The residues that formed the binding pockets were identified through an extensive literature search [24]. As shown in Table 1, the residues that constituted the tyrosinase binding sites were identified from the literature. Based on this evaluation, relevant data were gathered to identify the key atoms involved in binding.

### *In silico* molecular docking of tyrosinase inhibitors

The strength of the interaction between a ligand and a target can be predicted using the widely used docking research tool, AutoDock Vina. One of the most significant interactions that AutoDock Vina can predict during the docking process is hydrogen bonding. The algorithm identifies and aligns the hydrogen bond donors and acceptors in the ligand and receptor molecules. This information is then used to predict the strength and stability of the hydrogen bonds that will form between them. The distance and orientation of the hydrogen bond donors and acceptors in the ligand and receptor molecules are calculated; these data are then used to predict the strength and stability of the hydrogen bonds that will form between them. Table 2 presents the shortest distances between the ligands (nuciferine, resorcinol, kojic acid, and ascorbic acid) and the target proteins. Table 3 indicates that nuciferine had the highest binding energy with TRP1 (5M8L) at –7.3 kcal/mol and with AbTYR (2Y9W) at –7.0 kcal/mol.

### 2D and 3D interactions of docked complexes of tyrosinase inhibitors

Biovia Discovery Studio, a powerful software package for molecular modeling, provides the ability to visualize and analyze both 2D and 3D molecular interactions. This program allows users to interactively explore and manipulate chemical structures, thereby facilitating the identification and visualization of 2D interactions such as hydrogen bonds, hydrophobic bonds, electrostatic interactions, and pi-pi stacking.

**Fig. 8.**
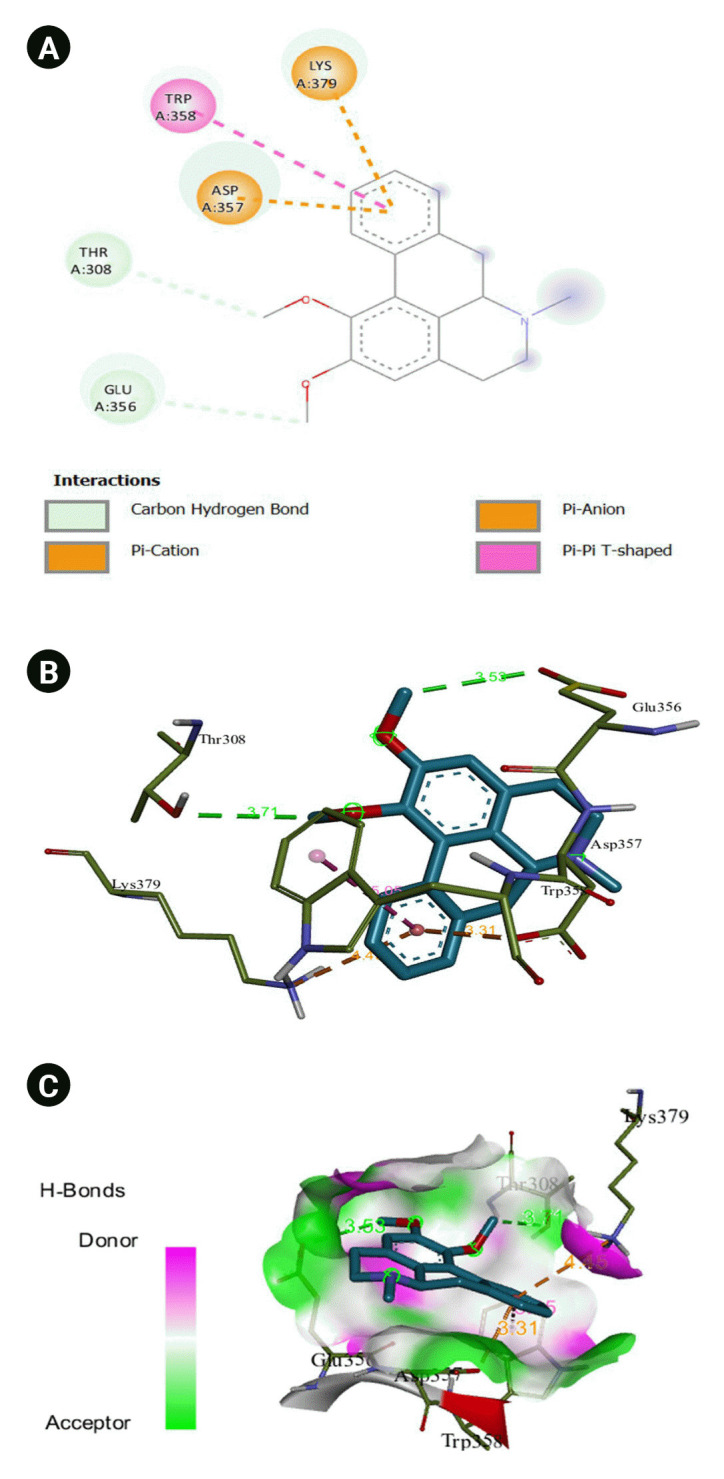
(A) 2D interaction of [2Y9W] AbTYR and nuciferine. (B) 3D interaction of [2Y9W] AbTYR and nuciferine. (C) AbTYR and nuciferine graphical representation.

**Fig. 9.**
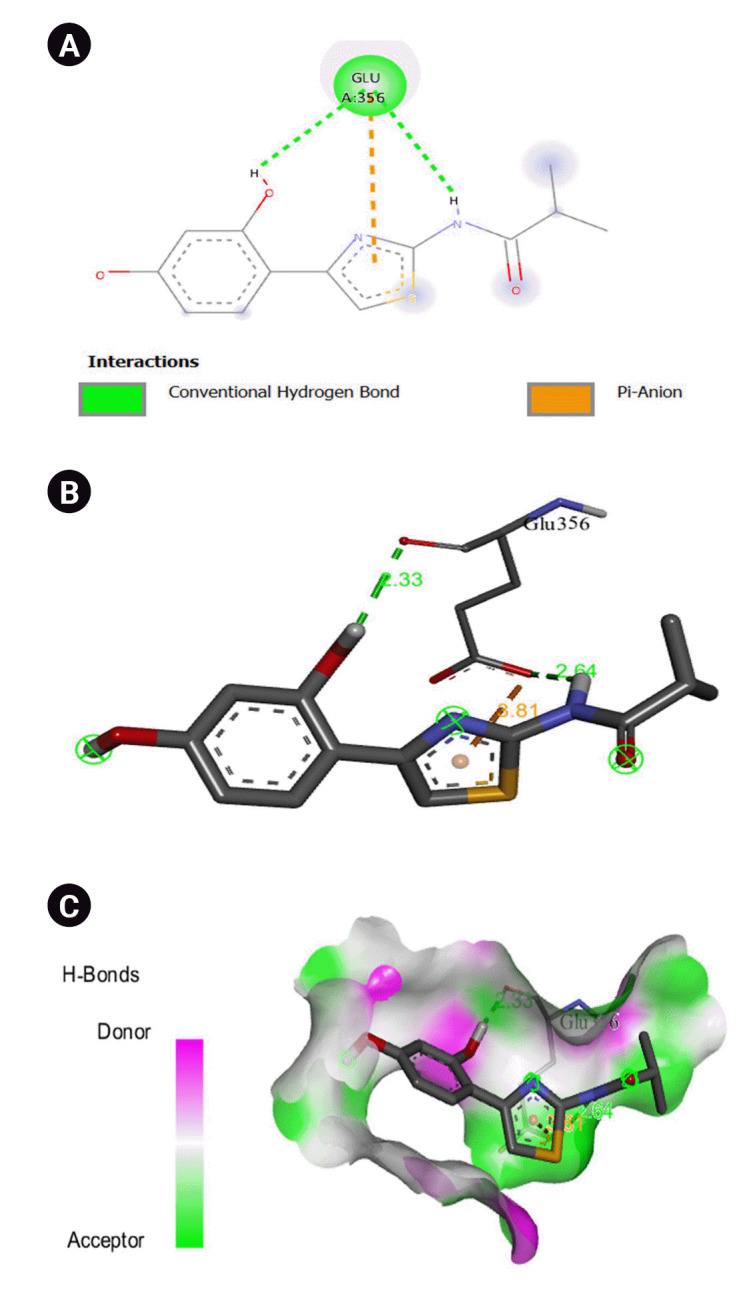
(A) 2D interaction of [2Y9W] AbTYR and thiazolyl resorcinol. (B) 3D interaction of [2Y9W] AbTYR and thiazolyl resorcinol. (C) H-bonds interaction of [2Y9W] AbTYR and thiazolyl resorcinol graphical representation.

**Fig. 10.**
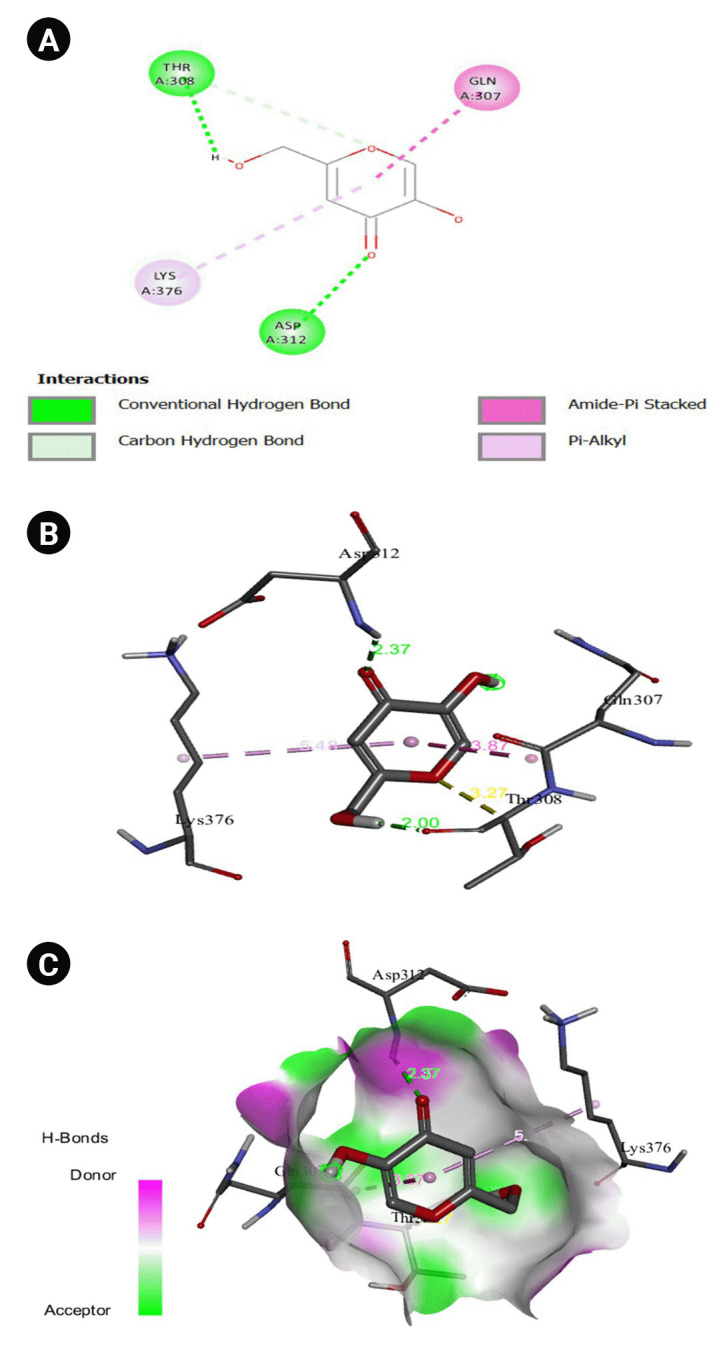
(A) 2D interaction of [2Y9W] AbTYR and kojic acid. (B) 3D interaction of [2Y9W] AbTYR and ascorbic acid. (C) H-bonds interaction of [2Y9W] AbTYR and ascorbic acid graphical representation.

**Fig. 11.**
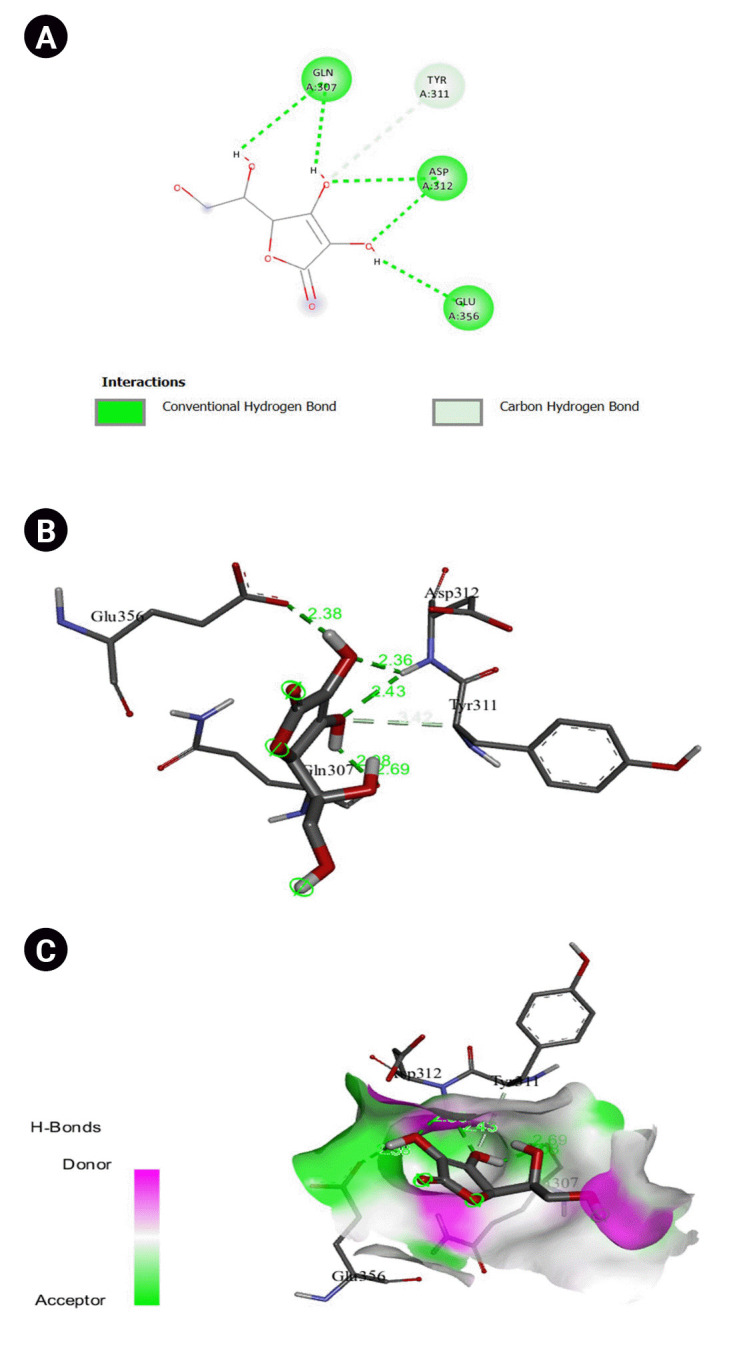
(A) 2D interaction of [2Y9W] AbTYR and ascorbic acid. (B) 3D interaction of [2Y9W] AbTYR and ascorbic acid. (C) H-bonds interaction of [2Y9W] AbTYR and ascorbic acid graphical representation.

**Fig. 12.**
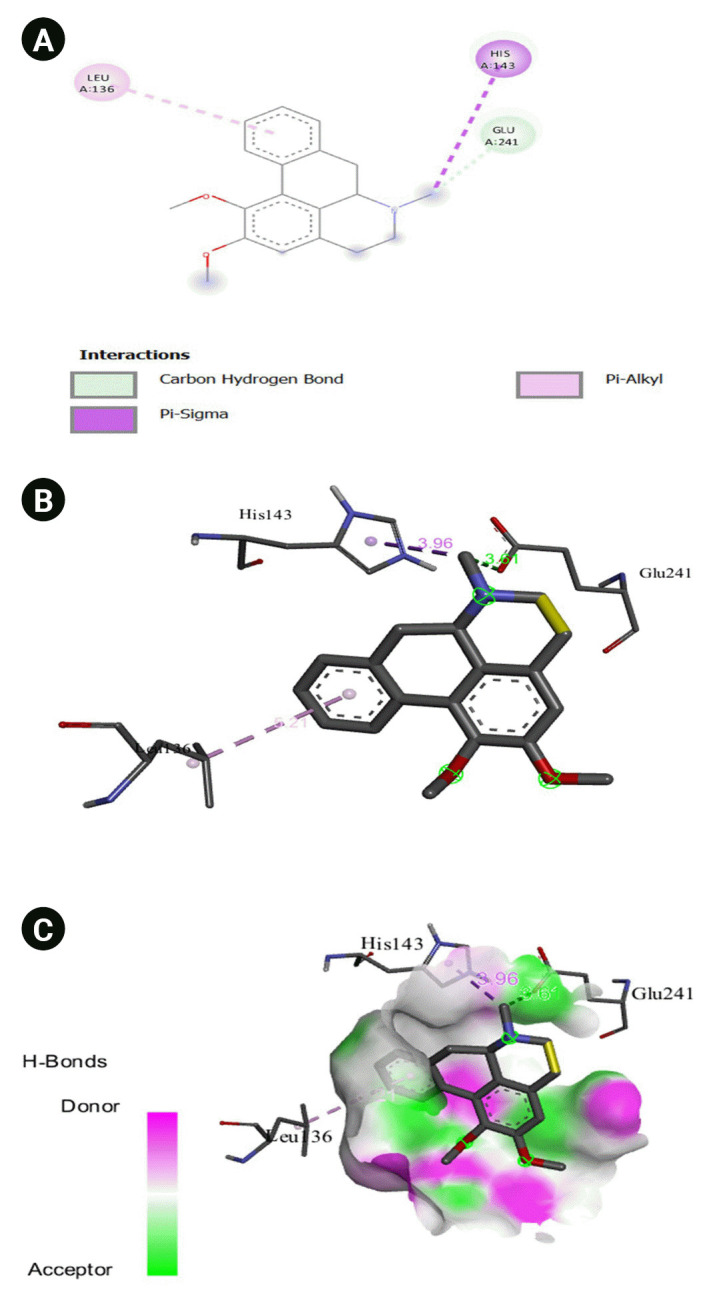
(A) 2D interaction of [5M8L] human TYR and nuciferine. (B) 3D interaction of [5M8L] human TYR and nuciferine. (C) H-bonds interaction of [5M8L] human TYR and nuciferine graphical representation.

**Fig. 13.**
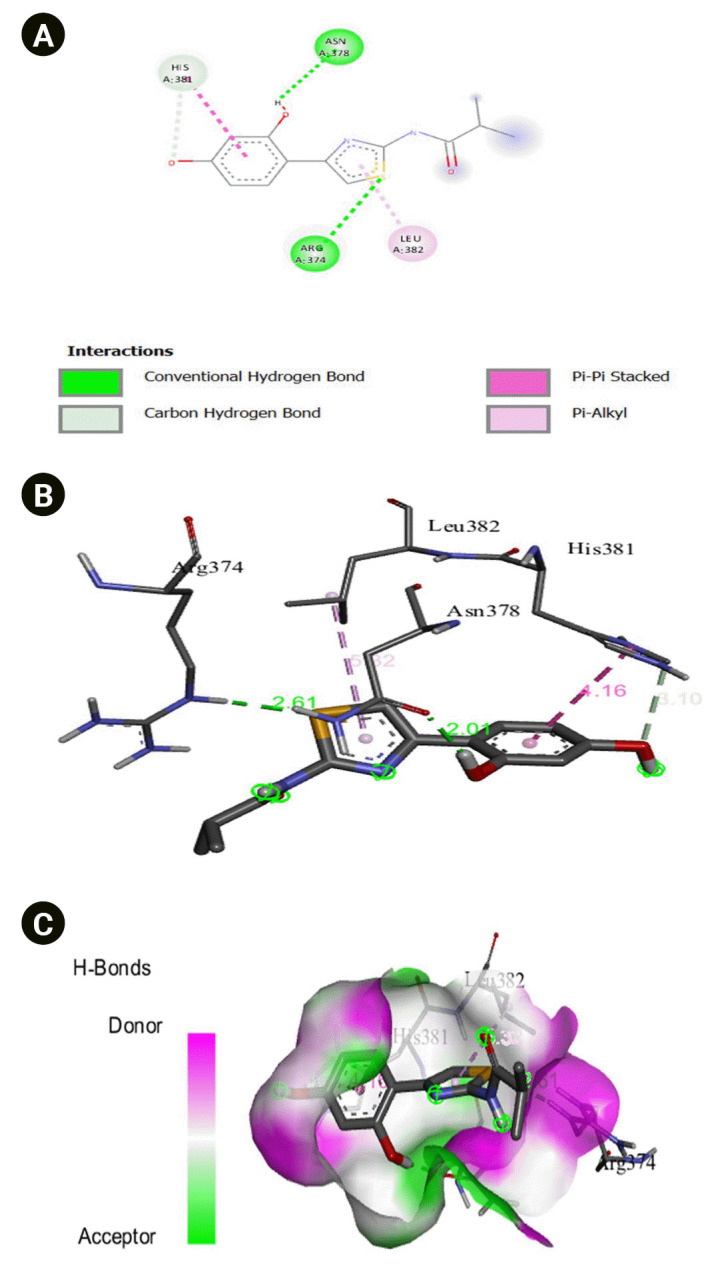
(A) 2D interaction of [5M8L] human TYR and thiazolyl resorcinol. (B) 3D interaction of [5M8L] human TYR and thiazolyl resorcinol. (C) H-bonds interaction of [5M8L] human TYR and thiazolyl resorcinol graphical representation.

**Fig. 14.**
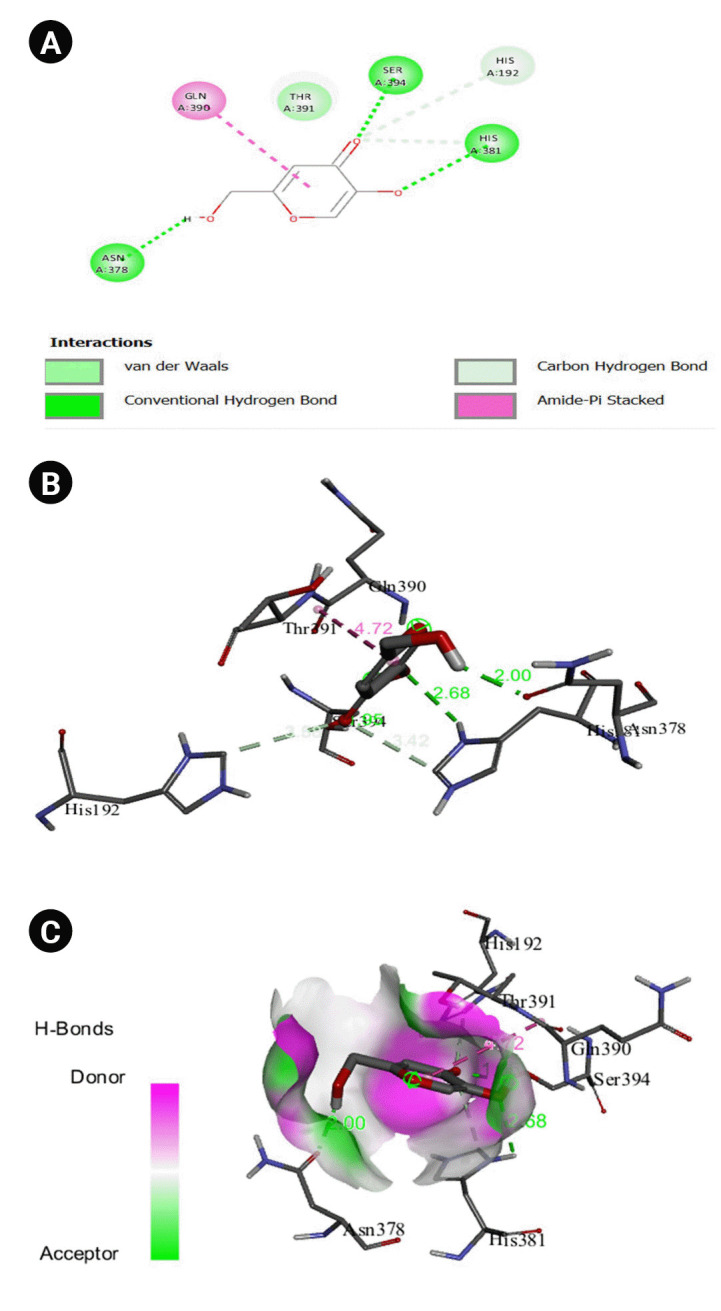
(A) 2D interaction of [5M8L] human TYR and kojic acid. (B) 3D interaction of [5M8L] human TYR and kojic acid. (C) H-bonds interaction of [5M8L] human TYR and kojic acid graphical representation.

**Fig. 15.**
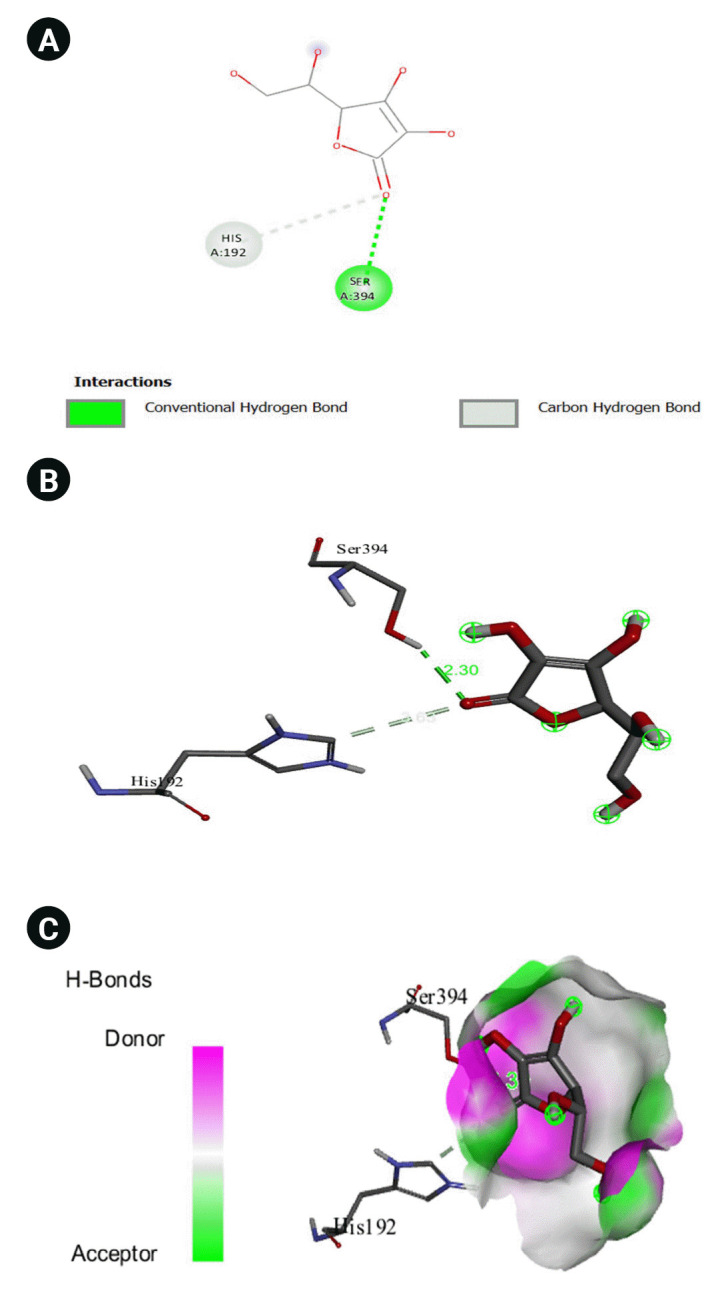
(A) 2D interaction of [5M8L] human TYR and ascorbic acid. (B) 3D interaction of [5M8L] human TYR and ascorbic acid. (C) H-bonds interaction of [5M8L] human TYR and ascorbic acid graphical representation.

The 2D interaction between [2Y9W] AbTYR and nuciferine is visualized in Fig. 8A. Fig. 8B provides a 3D representation of this interaction. The hydrogen bond interactions between these two entities are graphically represented in Fig. 8C. The interaction between [2Y9W] AbTYR and thiazolyl resorcinol is depicted in a 2D format in Fig. 9A, while Fig. 9B offers a 3D representation of the same. The hydrogen bond interactions between them are graphically illustrated in Fig. 9C. Fig. 10A visualizes the 2D interaction between [2Y9W] AbTYR and kojic acid. A 3D representation of this interaction is provided in Fig. 10B. The hydrogen bond interactions between these two are graphically represented in Fig. 10C. The 2D interaction between [2Y9W] AbTYR and ascorbic acid is analyzed in Fig. 11A. Fig. 11B offers a 3D visualization of this interaction. The hydrogen bond interactions between them are graphically represented in Fig. 11C. Fig. 12A depicts the 2D interaction between [5M8L] human TYR and nuciferine. The 3D interaction between these two is visualized in Fig. 12B. The hydrogen bond interactions between them are graphically illustrated in Fig. 12C. The 2D interaction between [5M8L] human TYR and thiazolyl resorcinol is investigated in Fig. 13A. Fig. 13B provides a visualization of the three-dimensional interaction between these two. The hydrogen bond interactions between them are graphically represented in Fig. 13C. Fig. 14A explores the 2D interaction between [5M8L] human TYR and kojic acid. The 3D interaction between these two is examined in Fig. 14B. The hydrogen bond interactions between them are graphically illustrated in Fig. 14C. The 2D interaction between [5M8L] human TYR and ascorbic acid is explored in Fig. 15A. Fig. 15B examines the 3D interaction between these two. The hydrogen bond interactions between them are graphically represented in Fig. 15C.

## Discussion

Mushroom tyrosinase is often used as a model in the study of the biochemical and enzymatic properties of tyrosinase. This is due to its relative ease of acquisition and use in laboratory environments compared to human tyrosinase. Such comparisons have yielded significant insights into the structure, function, and mechanisms of tyrosinase enzymes more broadly. Despite the lack of resolved crystal structures for full-length human tyrosinase, it is often compared with mushroom tyrosinase in scientific research. This comparison is largely driven by their similar enzymatic functions and structures, coupled with the availability of crystallographic data for mushroom tyrosinase.

Although the full structure of human tyrosinase has not been definitively determined, a comparison with mushroom tyrosinase provides a valuable method for gaining insights into its structure and function. The similarities between these two enzymes, in both function and structure, enable us to make informed assumptions and predictions. These can assist in understanding the role of human tyrosinase in melanin synthesis and associated diseases.

The active site pocket of the tyrosinase catalytic center is composed of two Cu^2+^ ions and six histidine residues. These residues include His61, His85, His94, His259, His263, and His296, as found in the *Agaricus bisporus* mushroom tyrosinase (PDB ID 2Y9W) [6,25].

Kojic acid has been reported to exhibit a binding energy of –5.4 kcal/mol with *Agaricus bisporus* tyrosinase (PDB ID 2Y9X) [26]. This is slightly higher than our docking result, which showed a binding energy of –5.2 kcal/mol with the same tyrosinase (PDB ID 2Y9W). In contrast, ascorbic acid demonstrated a binding energy of –5.4 and 5.9 kcal/mol with TRP1 (PDB ID 5M8L) and *Agaricus bisporus* tyrosinase (PDB ID 2Y9W) respectively [27].

Ascorbic acid has been identified as a potent tyrosinase inhibitor, exhibiting hydrophobic interactions with the amino acid residues Phe264, His263, Ser282, and Val283. It also forms two hydrogen bonds with Cu^2+^(1) and Cu^2+^ (2), at distances of 3.57 and 3.41 Å, respectively [28]. The aromatic ring of resorcinol is reported to engage in a pi-pi interaction with His367 and a hydrophobic interaction with the blocking residue Val377 in the TRP1 active site [29]. These pi-pi interactions can be observed in the 2D interactions of [2Y9W] AbTYR-nuciferine, [2Y9W] AbTYR-thiazolyl resorcinol, [5M8L] human TYR-nuciferine, and [5M8L] human TYR-thiazolyl resorcinol [29]. It has been reported that the binding of a hydroxide anion with copper ions significantly alters the active site of tyrosinase, leading to an increase in binding energy [25]. This was also observed in our molecular docking screening, where hydroxide ion interactions were noted with nuciferine, kojic acid, and ascorbic acid. Given these findings, the design of highly potent inhibitors against tyrosinase should aim to satisfy both the affinity for copper ions and the charge neutrality of the entire molecule [25].

Melanocytes, which are located in the basal layer of the epidermis, are responsible for skin pigmentation. They produce and transfer mature melanosomes to keratinocytes. The enzyme TYR plays a crucial role in melanin production, acting as the primary rate-limiting step. Melanin binds to nuciferine, along with TRP1 and AbTYR, with a binding energy greater than -5.0 kcal/mol. This binding energy is compared to that of kojic acid, ascorbic acid, and isobutylamido thiazolyl resorcinol. Nuciferine exhibits a higher binding energy than other drugs. Natural synthetic inhibitors are generally favored over purely synthetic inhibitors [30]. Nuciferine has significant market potential in the Ayurvedic-based cosmetic industry. To determine pharmacologically active concentrations, further *in vitro* tyrosinase studies and *in vivo* pigmentation models will be conducted. After toxicity evaluation on keratinocytes and fibroblasts, dermal permeability will need to be assessed for potential formulation into a dermal delivery substance.

## Figures and Tables

**Table 1.** Details of the active site residues used for molecular docking studies for the TRP1 and AbTYR proteins

Target protein	Active site residues	Grid size (x, y, z in Å)	Grid center (x, y, z) coordinates
TRP1	HIS 192, HIS 215, HIS 224, HIS 377, HIS 381, HIS 404	48 × 38 × 48	36.125, 140.659, 214.023
AbTYR	HIS56, LYS63, LEU12, MET319, PRO12, GLU317, ILE55, ASN57, GLN331, SER337, VAL150, VAL332, ILE328, PHE148, ILE96, PHE105, TYR78, TYR98, HIS76, GLU139	30 × 30 × 30	–6.694, 45.965, 85.063

**Table 2.** Hydrogen bond distance in multiple molecular targets of melanin: nuciferine, resorcinol, kojic acid and ascorbic acid

Target	PDB ID	Nuciferine		Resorcinol		Kojic acid		Ascorbic acid	
		Hydrogen bond residues	Distances of hydrogen-bonds	Hydrogen bond residues	Distances of hydrogen-bonds	Hydrogen bond residues	Distances of hydrogen-bonds	Hydrogen bond residues	Distances of hydrogen-bonds
AbTYR	2Y9W	GLU356	3.53	GLU356	2.33	THR308	2.00	GLN307	2.08
Human tyrosinase	5M8L	GLU241	3.67	ASN378	2.01	SER394	1.95	SER394	2.30

PDB, Protein Data Bank.

**Table 3.** Docking analysis of nuciferine and vemurafenib on multiple molecular targets of melanoma

Target	PDB ID	Nuciferine			Resorcinol			Kojic acid			Ascorbic acid		
		kcal/mol	No. of H-bond	H-bonds’ binding residues	kcal/mol	No. of H-bond	H-bonds’ binding residues	kcal/mol	No. of H-bond	H-bonds’ binding residues	kcal/mol	No. of H-bond	H-bonds’ binding residues
AbTYR	2Y9W	–7.0	2	GLU356, THR308	–5.9	1	GLU356	–5.2	2	THR308, ASP312	–5.4	4	TYR311, GLN307, GLU356, ASP312
Human tyrosinase	5M8L	–7.3	1	GLU241	–6.5	3	ASN378, ARG374, HIS381	–5.8	5	SER394, HIS381, HIS192, THR391, ASN378	–5.9	2	HIS192, SER394

PDB, Protein Data Bank; H-bond, hydrogen bond.
